# The Diagnostic and Therapeutic Potential of Galectin-3 in Cardiovascular Diseases

**DOI:** 10.3390/biom12010046

**Published:** 2021-12-29

**Authors:** Grażyna Sygitowicz, Agata Maciejak-Jastrzębska, Dariusz Sitkiewicz

**Affiliations:** Department of Clinical Chemistry and Laboratory Diagnostics, Medical University of Warsaw, 02-097 Warsaw, Poland; agata.maciejak@wum.edu.pl (A.M.-J.); dariusz.sitkiewicz@gmail.com (D.S.)

**Keywords:** galectin-3, cardiac fibrosis, heart failure, atrial fibrillation, chronic inflammation, MMPs, microRNAs, lncRNAs

## Abstract

Galectin-3 plays a prominent role in chronic inflammation and has been implicated in the development of many disease conditions, including heart disease. Galectin-3, a regulatory protein, is elevated in both acute and chronic heart failure and is involved in the inflammatory pathway after injury leading to myocardial tissue remodelling. We discussed the potential utility of galectin-3 as a diagnostic and disease severity/prognostic biomarker in different cardio/cerebrovascular diseases, such as acute ischemic stroke, acute coronary syndromes, heart failure and arrhythmogenic cardiomyopathy. Over the last decade there has been a marked increase in the understanding the role of galectin-3 in myocardial fibrosis and inflammation and as a therapeutic target for the treatment of heart failure and myocardial infarction.

## 1. Introduction

Galectins are a family of glycan-binding proteins, which were named and classified in 1994 considering their affinity for β-galactosides and significant sequence similarity in the carbohydrate-binding domains (CRDs) [1,2]. To date, 16 protein-coding galectin genes have been identified including 12 members in human tissues encoding galectins 1, 2, 3, 4, 7, 8, 9, 10, 12, 13, 14 and 16 [2,3,4]. Galectins 5 and 6 are present in rodents, while galectins 11 and 15 are found in sheep and goats [2].

Galectins are synthesized in cytosol but they can be secreted from the cells in not yet fully elucidated mechanisms. Functioning both outside and inside the cell—galectins participate in various cell processes, including transport of glycoprotein vesicles, chemotaxis, proliferation, pre-RNA splicing and apoptosis. Extracellular galectins can act through binding to cell surface glycans, while intracellular effectors, mediating galectin function, still remain, to a great extent, unknown.

The members of the galectin family are classified into three types according to their molecular architecture: (1) prototype, which are usually homodimers containing one carbohydrate recognition domain in each subunit; (2) tandem, which are monomers containing two CRDs joined by a linker sequence; (3) chimeric, containing C-terminal CRD joined to a large repeating sequence and N-terminal domain [2].

## 2. Galectin-3 Structure and Functions

Galectin-3 (Gal-3) is widespread and present in various organs including: the lungs, heart, stomach, colon, adrenals, uterus and ovaries [5]. Galectin-3 is the only galectin of chimeric type in the galectin family. Galectin-3, also known as a binding protein for IgE, Mac2, CBP30 and CP35, is encoded by the *LGALS3* gene present on chromosome 14, locus q21-22. It consists of six exons and five introns involving about 17 kilobases. Galectin-3 expression is regulated by promotor methylation status of *LGALS3*, and such elements as: CRE motifs, region similar to nuclear factor kappa B (NF-κB), GC regions located in galectin-3 promoter [6]. The galectin-3 gene contains also a special regulatory element called galig (galectin-3 internal gene) located in the second intron of the *LGALS3* gene [7].

Galectin-3 consists of 251 amino acid residues of relative molecular mass 29–35 kDa and it has been identified for the first time in murine peritoneal macrophages [1]. It contains three domains: (1) short N-terminal constituting a unique region of 12 amino acids and containing a site of serine 6 phosphorylation for controlling its nuclear location and abolishing its affinity to ligand [8,9]; (2) a 100-amino acid sequence similar to collagen, containing proline, glycine and tyrosine tandem repetitions and containing a fissionable domain of collagenase H, in which histidine 64 is the site of action of matrix metalloproteinases (MMPs), such as MMP-9 and MMP-2; (3) spherical C-terminal CRD containing an Asp-Trp-Gly-Arg motif (NWGR), similar to those described in the anti-apoptotic protein Bcl-2 [1].

Galectin-3 can form dimers or pentamers in specific circumstances, when galectin-3 concentration is high or when ligands are present [10]. Monomeric galectin-3 undergoes physicochemical modifications, which increase the range of its biological functionality, particularly extracellular activity. The most important mechanisms leading to galectin-3 “bioactivation” is multimerization and formation of galectin-3 lattice. Galectin-3 has various functions, depending on its cellular location (Table 1).

**Figure 1:**
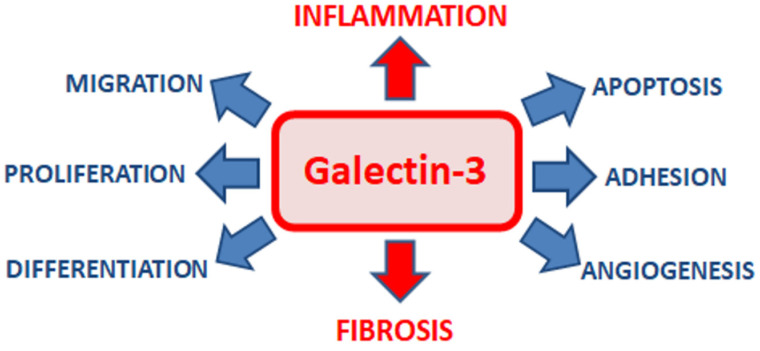
Biological functions of galectin-3.

Galectin-3 participates in various pathophysiological processes, including apoptosis [6], adhesion [16], angiogenesis [17], cell migration [17], proliferation [16], and differentiation [18], but its main function is induction of inflammatory condition and fibrotic process [6] (Figure 1).

## 3. Galectin-3 in Cardiovascular Diseases

**Figure 2:**
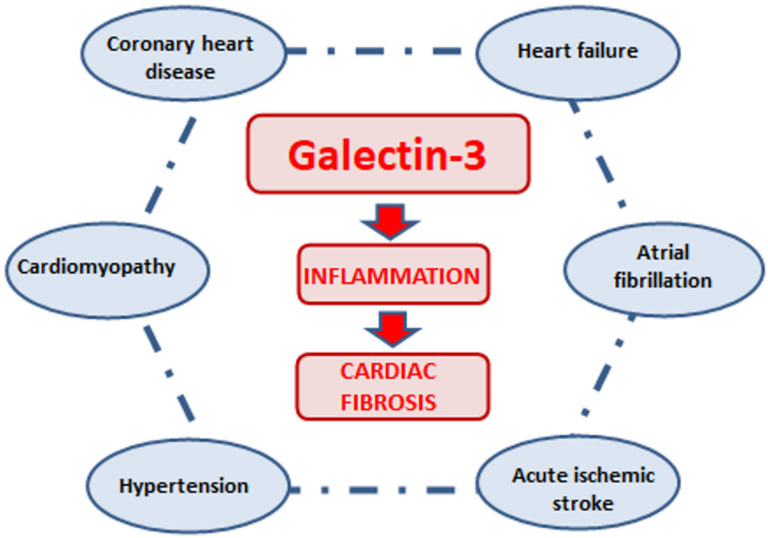
Galectin-3 and cardiovascular diseases.

In view of its multidirectional activity, galectin-3 plays an important role in many various clinical conditions and disease entities. Increased galectin-3 expression has been documented in cardiovascular diseases (CVDs), such as atherosclerosis, acute ischemic stroke, acute coronary syndrome (ACS) and heart failure (HF), arterial hypertension, cardiomyopathies or atrial fibrillation (AF) [19] (Figure 2).

### 3.1. Atherosclerosis

Atherosclerosis is a complex inflammatory process, initiated by changed permeability of arterial wall cells and focal subendothelial accumulation of LDL lipoproteins, leading to formation of atheromatous plaques characterised by inflammatory condition and increased oxidative stress [20,21]. The later inflammatory reaction includes a massive participation of monocytes and macrophages changing the structure of the vascular wall [10,22]. Endothelial cells become activated and vascular smooth muscle cells (VSMCs) actively proliferate to produce extracellular matrix. Over the years, the participation of many inflammatory condition markers in the atherosclerotic process has been studied [21,22,23,24,25] and then a potential role of galectin-3 as a mediator of atherosclerosis has been suggested. Many studies have shown that galectin-3 contributes to macrophage differentiation [26], formation of foam cells [27], endothelial dysfunction [27,28] and VSMC proliferation and migration in atherogenesis [29]. The amplification of cardiovascular system inflammatory condition and accumulation of lipids in macrophages caused by galectin-3 are the most important mechanisms of atherosclerosis development, which are stimulated by local or circulating galectin-3.

Study performed by Ou et al. [30], using human umbilical vein endothelial cells (HUVECs), showed that exogenous addition of galectin-3 and oxidised low-density lipoprotein (oxLDL) to cell cultures increased the expression of lectin-like oxLDL receptor 1 (LOX-1) and promoted endothelial dysfunction via LOX-1/ROS/p38/NF-kB-mediated signalling pathway. It has been suggested by authors that galectin-3 enhances LOX-1 expression and induced pro-inflammatory response [30].

### 3.2. Acute Myocardial Infarction

Galectin-3 has been described as a factor contributing to the development and destabilisation of atheromatous plaques through propagation of inflammatory condition, interaction with lipopolysaccharides (LPSs) and promotion of VSMC phenotypic transformation [31]. Therefore, galectin-3 can be involved in the pathogenesis of ACSs caused by atherosclerosis. A significantly increased galectin-3 expression has been demonstrated in the early phase of acute myocardial infarction (AMI) and ACS [32,33]. In in vivo studies, using recombinant galectin-3 in rat experimental model, it has been observed higher collagen deposition and thick collagen content in the infarct region. This study presented potential application of exogenous galectin-3 to assess the myocardial remodelling process after MI [34]. Both experimental and clinical studies have demonstrated that galectin-3 is an independent predictor of mortality for any cause, death for cardiovascular causes and development of HF [35,36]. In the later phase of ACS, galectin-3 enhances the transition from acute to chronic inflammatory condition and causes myocardial fibrosis, leading to unfavourable ventricular remodelling [35].

### 3.3. Heart Failure

In recent decades, cardiac remodelling and myocardial fibrosis have been accepted as the main heart failure-inducing factors. Galectin-3, exogenously added, is closely related to myocardial fibrosis and is strongly expressed in cardiac myofibroblasts, which can be used as an independent predictor of myocardial fibrosis [37]. Sharma et al. [37] revealed that recombinant galectin-3, exogenously injected into the pericardial sac of healthy rats over a long term, led to LV dysfunction and deterioration of cardiac function. Galectin-3 induced collagen deposition and fibroblast proliferation. Liu et al. [38] demonstrated that when the transforming growth factor β (TGFβ)/Smad3 signalling protein pathway was inhibited by N-acetyl-seryl-aspartyl-lysyl-proline (Ac-SDKP), the expression of profibrotic and inflammatory factors induced by intrapericardial infusion of galectin-3 was significantly reduced and cardiac remodelling and dysfunction were less pronounced. That fact suggests that galectin-3-induced myocardial remodelling may be associated with an activation of TGFβ/Smad3 signal transduction pathway. On the other hand, another experiment [39] has proved that excessive galectin-3 expression favours type I collagen synthesis, while galectin-3 secretion inhibition reduces the synthesis and deposition of that collagen.

### 3.4. Hypertrophic Cardiomyopathy

Hypertrophic cardiomyopathy (HCM), a primary myocardial disease, is characterised by myocardial hypertrophy. The development of cardiac fibrosis and irreversible change of ventricular structure significantly contribute to sudden cardiac death in patients with HCM [40]. In their case-control study, Yakar et al. [41] analysed and compared the relationship between serum galectin-3 concentration and left ventricular (LV) function in 40 patients with HCM and in 35 age-matched healthy volunteers. That study demonstrated that concentration of serum galectin-3 was significantly elevated in HCM patients compared with the control group. Furthermore, levels of serum galectin-3 were positively correlated with interventricular septum thickness and LV mass index. The concentration of serum galectin-3 was, however, not related to the degree of LV outflow tract obstruction. For that reason, it was accepted that serum galectin-3 concentration was related to the degree of LV hypertrophy but not to the LV diastolic and systolic dysfunction [41]. However, whether galectin-3 plays a major causative role in discussed process is not completely justified because there are studies showing that up-regulation of cardiac galectin-3 is not a critical disease modulator of cardiomyopathy induced by β_2_-adrenoceptor over-expression [42]. These findings raise a possibility that role of galectin-3 in the development of cardiomyopathy might not be universal but rather dependent to disease aetiology. On the other hand, another experiment [43] has revealed that galectin-3 is not a critical mediator of the fibrotic cardiomyopathy associated with pressure overload. Myocardial galectin-3 expression did not affect the survival, systolic and diastolic dysfunction, and cardiac fibrosis.

### 3.5. Atrial Fibrillation

Atrial fibrillation (AF) is a cardiac arrhythmia characterised by a rapid and irregular heart rate and is the most frequent and most serious arrhythmia. AF is closely related to high mortality rate, cerebral stroke and HF [44]. Particularly, atrial interstitial fibrosis seems to be the key factor contributing to AF development [45]. It is known that left atrial (LA) interstitial fibrosis plays a significant role in initiating and maintaining atrial fibrillation [45,46].

Galectin-3 enhances cardiac fibrosis and remodelling and is a well-documented cause of arrhythmia. Most studies have focused on the relationship between levels of serum galectin-3 and cardiac fibrosis in HF. The role of galectin-3 in the pathophysiology of atrial fibrillation has not been fully elucidated as yet. Sonmez et al. [47] were the first to study the levels of new biomarkers of inflammation in serum in patients with atrial fibrillation compared with those with sinus rhythm. The results of the study demonstrated that the levels of the new circulating markers of remodelling, such as galectin-3, MMP-9 and PIIINP (amino terminal peptide of type III procollagen) were significantly higher in patients with atrial fibrillation. Moreover, galectin-3, MMP-9 and PIIINP concentrations in serum were strongly positively correlated with LA volume and LA volume index [47]. The observational study by Gurses et al. [48] demonstrated also that serum galectin-3 concentration and LA volume index were significantly higher in patients with atrial fibrillation, which could have suggested that concentration of serum galectin-3 in AF can be correlated with atrial remodelling. The study also demonstrated that serum galectin-3 concentration was significantly higher in patients with persistent atrial fibrillation than in those with paroxysmal atrial fibrillation [48]. The study conducted by Yalcin et al. [49] revealed that the LA volume index and serum galectin-3 concentration were independently correlated with the range of LA fibrosis detected by means of delayed-enhancement magnetic resonance imaging (DE-MRI) in patients with paroxysmal atrial fibrillation with preserved LV function. These results suggest that concentration of serum galectin-3 is significantly correlated with atrial remodelling in patients with paroxysmal atrial fibrillation with preserved LV function [49].

### 3.6. Arterial Hypertension

Arterial hypertension is usually a chronic pathological condition characterised by elevated arterial pressure. Hypertensive cardiac remodelling starts with inflammation, increased deposition of extracellular matrix proteins leading then to myocardial fibrosis and, finally, to heart dysfunction [50]. It has been demonstrated that serum galectin-3 concentration increases in hypertensive patients, but that phenomenon is more pronounced in patients with LV hypertrophy. Therefore, galectin-3, as an important biomarker of early cardiac remodelling, is independently correlated with left ventricular remodelling (LVR). Moreover, LV mass was independently correlated with concentration of serum galectin-3 in hypertensive patients [51].

Primary aldosteronism is regarded as the most frequent cause of secondary arterial hypertension. Patients with primary aldosteronism show an increased susceptibility to heart muscle inflammation and fibrosis [52]. It has been demonstrated that aldosterone can induce galectin-3 secretion [50]. Galectin-3 is one of the most important mediators between macrophage activation and myocardial fibrosis. In a prospective pilot clinical study, serum galectin-3 concentration was significantly higher in the group of patients with aldosterone-producing adenoma. Moreover, both the degree of myocardial fibrosis and serum galectin-3 concentration returned to normal after adrenalectomy [53]. Azibani et al. [54] observed for the first time that hyperaldosteronism increased the number of inflammatory factors, including galectin-3, and accelerated the hypertension-induced fibrosis. Many studies in this aspect were conducted. However, the role of galectin-3 in hyperaldosteronism-induced inflammation remains unclear [54].

### 3.7. Acute Ischemic Stroke (AIS)

AIS is a cerebral tissue infarction caused by occlusion of cerebral arteries, with injury of the neurons, astrocytes and oligodendrocytes. It is the most important vascular event in the central nervous system, leading to death or disability. Dong et al. [55] assessed the role of galectin-3 in patients with AIS. They demonstrated that serum galectin-3 concentration was significantly higher in AIS patients compared with healthy individuals, what increased the intensity of AIS and infarction volume. Besides that, higher levels of serum galectin-3 were independently correlated with increased risk of death, significant disability, recurrent stroke and vascular events [56,57]. In in vitro studies, performed using human cortical neuronal (HCN) cell lines culture, a knockdown of galectin-3 expression with siRNA dramatically increased neuronal cell viability and simultaneously reduced apoptosis and serum levels of proinflammatory cytokines, including interleukin-1, -6 (IL-1, -6) and NF-κB and also caspase-3, a protein associated with apoptosis [55]. As a result of ischaemia, galectin-3 is released by microglia in the injured cerebral tissue. Then, the released galectin-3 contributes to microglia activation through binding to toll-like receptor 4 (TLR4), what causes an exacerbation of the inflammatory response [58]. Furthermore, the effect of serum galectin-3 concentration of AIS patients may be associated with serum lipid concentration regulation. AIS is reversely proportionally related to HDL cholesterol concentration, which, in turn, is reversely related to mortality from ischemic stroke and vascular events [59]. Earlier studies have demonstrated a reverse relationship between galectin-3 and HDL-C in serum, what suggests that AIS regulation, in which galectin-3 participates, is possibly associated with dyslipidaemia and inflammatory condition [60,61]. In a prospective cohort study involving 2970 patients with AIS, increased galectin-3 and decreased HDL-C levels of serum were observed, what could have intensified inflammatory condition and oxidative stress after ischemic stroke [57]. Moreover, it seems that concentrations of serum galectin-3 and HDL-C exerta combined effect on AIS prognosis.

Furthermore, a neuroprotective effect in cerebral stroke has been described. Galectin-3 is of key importance for activation, migration and proliferation of microglia after ischemic stroke [62]. Galectin-3 deficiency is associated with a significant increase of the size of ischemic lesions and number of apoptotic neurons. Microglial activation and proliferation mediated by galectin-3 are associated with Gal-3/IGF-R1 (insulin-like growth factor receptor 1) interaction in response to ischemic injury [62]. The data suggest that galectin-3 plays a neuroprotective role in injured brain.

In summary, galectin-3 can simultaneously exert a negative effect and show a neuroprotective action in AIS. One of the possible causes is that circulating galectin-3 serum levels are associated with various stages of AIS and a coincidence of two unfavourable effects or the neuroprotective role are not necessarily associated with the circulating galectin-3.

## 4. Diagnostic Usefulness of Serum Galectin-3 Concentration

The inflammatory process and especially its acute phase revealed as a result of bacterial infection and in certain types of tumours, is closely related to the circulating neutrophils. The recruitment of the cells is a characteristic feature of acute inflammation and they are the first cells to migrate towards the inflammation site. Galectin-3 is strongly involved in the modulations of inflammatory processes and disorders underlying the inflammatory condition. The proinflammatory effect of galectin-3 is associated with activation of NF-kB transcription factor, induction of tumour necrosis factor α (TNF-α) and interleukin 6, regulation of cell adhesion, promotion of cell activation and chemotaxis and regulation of cell growth or apoptosis [63].

Inflammatory condition underlies many atherosclerosis-related diseases. Galectin-3 seems to be an important and, at the same time, useful biomarker of atherosclerosis and its special role has been observed in the process of atheromatous plaque destabilisation. A positive relationship has been demonstrated between serum galectin-3 concentration and the number and calcification area of atheromatous plaques [64]. Moreover, high concentrations of serum galectin-3 are a harbinger of clinical failures associated with higher risk of overall mortality or mortality due to cardiovascular reasons and HF [65]. The authors of another paper [66] have stressed that higher galectin-3 serum concentrations determined in patients on admission to hospital are associated with severe course of stroke and frequently with a poor prognosis at discharge from hospital. An unfavourable prognosis is frequently even poorer when a close relationship is present between levels of serum galectin-3 and markers of lipid metabolism [60], carbohydrate metabolism [67,68], renal function [69,70] or echocardiographic parameters serving for assessment of myocardial function and structure [71,72]. In individuals, in whom no metabolic disorders or excessive body mass or increased triglyceride and total cholesterol concentrations have been found, it has been demonstrated that the age itself can distinguish subjects with higher and lower serum galectin-3 concentrations (individuals <40 years of age: 11.5 (9.5–13.60) ng/mL vs. those aged ≥40 years: 12.4 (10.6–14.4) ng/mL) [73].

In the assessment of the cardiovascular risk of primary importance are disorders of lipid–carbohydrate profile or renal function. It was found [60] that in individuals aged <40 years after myocardial infarction, serum galectin-3 concentration was strongly positively related to non-HDL-cholesterol concentration and negatively related to HDL-cholesterol level. Patients with type 2 diabetes mellitus had higher serum galectin-3 concentrations than healthy individuals [67,68]. In patients with chronic and acute heart failure a relationship was observed between higher serum galectin-3 concentrations and renal failure parameters assessed by cystatin C or uric acid concentrations or by reduced estimated glomerular filtration rate (eGFR) [74]. The studies conducted by Mueller et al. [75] demonstrated that patients with eGFR >90 mL/min/1.73 m^2^ had lower levels of serum galectin-3 (median: 10.7 (9.3–12.4) ng/mL) compared with patients with eGFR <90 mL/min/1.73 m^2^, in whom higher levels of serum galectin-3 were found (median: 12.1 (10.2–14.1) ng/mL). The diverse clinical involvement of galectin-3 in many diseases is due to its role as a regulator of acute and chronic inflammation that links inflammation-related macrophages and the promotion of fibrosis. This makes galectin-3 not an organ-specific marker, but a marker specific to the pathogenesis of inflammatory and/or fibrotic disorders. This is because the primary sources of circulating galectin-3 are not always identifiable. A patient with heart disease may have varying degrees of inflammation and progression of the fibrotic process. Thus, the serum galectin-3 concentration may reflect different stages of the pathophysiological state. This is because the level of circulating galectin-3 in patients with different stages of heart disease does not differentiate between myocarditis and fibrosis and therefore it does not specifically reflect these conditions. Moreover, there are gender-related differences in serum galectin-3 concentrations. In women, higher serum galectin-3 concentrations were observed compared to men, as well as a stronger correlation between concentration of serum galectin-3 and other cardiovascular disease risk factors [61].

An increased secretion of galectin-3 promotes a release of inflammatory mediators, including TGF-β or interleukin 1 and 2, and also intensifies cardiac fibroblast proliferation. Activated cardiac fibroblasts are the main source of extracellular matrix (ECM) proteins, particularly collagen. Galectin-3 affects in the first place synthesis of type I collagen, leading to an impairment of the homeostasis between type I/III collagen content and thus to impairment of the systolic/diastolic function of the myocardium. These disorders contribute to progression of myocardial failure [76].

Galectins, apart from modulating inflammatory processes, play the dominant role in fibrotic processes. In heart failure the pathophysiological element of key importance is the progressing fibrosis of the myocardial tissue. In the myocardium, the level of galectin-3 expression is almost undetectable in cardiomyocytes, but in cardiac fibroblasts that lectin reaches a significantly higher concentration. Chronic kidney disease (CKD) is one of the risk factors of cardiovascular diseases, hence cardiac biomarkers play a significant role in the development of kidney diseases. An elevated serum galectin-3 concentration is associated with myofibroblast proliferation or intense fibrogenesis and may be a harbinger of kidney fibrosis process or even of CKD development [70].

In heart failure galectin-3 is released into extracellular space, promoting fibrotic process through activation of fibroblasts. The fibroblast activation is characterised by increased expression of cytoskeletal protein—alpha smooth muscle actin (α-SMA)—an intracellular fibrosis marker, and collagen type 1 (COL1α1)—an extracellular fibrosis marker. Both α-SMA and COL1α1 regulation in tissues affected by fibrosis is mediated by galectin-3. That process is mediated by activation of cyclin-dependent kinase inhibitor 1A (CDKN1A), inhibin beta A, fibronectin 1, as well as extracellular signal-regulated kinase (ERK) and phosphatidylinositol 3-kinase (PI3K). Thus, the central place in the regulation of fibrotic process development is taken by galectin-3, which participates in the regulation of expression of fibrotic matrix components (α-SMA and COL1α1) and in extracellular matrix turnover through a number of tissue inhibitors of metalloproteinases (TIMPs) and matrix metalloproteinases. Galectin-3 influences the development of myocardial fibrosis through an effect on the important intermediates of that regulation: phosphatase and tensin homolog (PTEN) and protein tyrosine kinase 2 (PTK2). Exerting an effect on PTEN, it inhibits MMP-14 activity, contributing to the development of fibrosis, and inhibits MMP-9 activity, preventing the cardiac fibrosis process. On the other hand, the promoting of myocardial fibrosis with participation of MMP-9 is mediated by PTK2 [77].

The effect of galectin-3 is closely related to the heart failure markers useful in clinical practice, namely natriuretic peptides. The studies conducted by Felker et al. [78] have confirmed the relationship between increased galectin-3 serum concentration and the intensity of heart failure in individual NYHA classes, monitored by NT-proBNP concentration. In other studies a positive correlation between serum galectin-3 concentration and a negative correlation between left ventricular ejection fraction (LVEF%) and the degree of heart failure progression (max. NYHA IV class) have been demonstrated. A relationship has also been found between plasma galectin-3 concentration and the change of the left ventricular structure and function, what has confirmed that galectin-3 can participate in the left ventricular remodelling process in patients with HF [79,80]. Moreover, the studies have confirmed a higher specificity of galectin-3 in predicting the occurrence and development of HF than that in the case of determination of NT-proBNP concentration alone. However, this has not been shown with respect to galectin-3 sensitivity in the prognosis of the occurrence and development of HF [80].

Our studies have unequivocally stressed the importance of galectin-3 as a predictor of death in one-year follow-up [81]. The research has also shown a positive correlation between concentrations of serum NT-proBNP and galectin-3 (r = 0.565, *p* = 0.035). In the studies conducted in patients with HF in NYHA III class [82] it has been observed that galectin-3 is an important predictor of the risk of death, taking into account the age and gender and also HF intensity (based on NT-proBNP concentration) and renal function disorders (acc. to eGFR value). In the studies by Tang et al. [83], increased plasma galectin-3 concentration was associated with advanced age and poor renal function. That correlation, revealed in a group of patients with chronic systolic heart failure, demonstrated that high plasma galectin-3 concentration was associated with renal failure and shorter survival of the patients.

On the other hand, Fermann et al. [84] demonstrated in their study higher serum galectin-3 concentrations in patients with myocardial failure and with confirmed renal dysfunction. It is worth to stress here, that in the case of heart or kidney failure it is recommended to analyse in serum galectin-3 concentration together with natriuretic peptide concentration values. A joint analysis of changes of these markers offers a possibility of a more accurate prognostication and, at the same time, confirmation of organ failure development.

A group of researchers [85] made an attempt to find a correlation between levels of serum galectin-3 and NT-proBNP and the inflammatory condition assessed by serum hsCRP concentration in patients with the first myocardial infarction, treated by percutaneous coronary intervention (PCI). In the paper by Szadkowska et al. [85] a detailed analysis of the above-mentioned markers in serum, depending on galectin-3 level, demonstrated three times higher NT-proBNP concentrations and two times higher hsCRP levels in patients presenting higher galectin-3 concentrations (>16 ng/mL), than in those with galectin-3 concentrations <16 ng/mL. In the paper, a positive correlation was also demonstrated between NT-proBNP and hsCRP concentrations (r = 0.45, *p* < 0.001) and galectin-3 and hsCRP concentrations (r = 0.20, *p* < 0.05).

In clinical practice, apart from commonly used heart failure markers—natriuretic peptides, increasingly frequently new tools are tested for the assessment of the degree of heart failure—and that concerns not only myocardial failure. They include, among other markers: GDF-15 (growth and differentiation factor-15) and ST2 (suppression of tumourigenicity 2), which belongs to the interleukin 1 family. GDF-15 is regarded as a prognostic marker in cardiovascular diseases and is frequently determined in combination with other prognostic factors, such as NT-proBNP or hsTnT. High GDF-15 concentrations were noted in hypertrophic and dilated cardiomyopathies, after volume overload, ischaemia and heart failure [86]. The ST2 receptor has two isoforms: membrane-bound receptor type 2 (ST2L) and soluble form (sST2), present in serum and most frequently used in diagnostic procedures [70,87]. The form sST2 is a “bait” for IL-33 and thus it counteracts its interaction with the membrane-bound ST2L, blocking the paracrine fibroblast-cardiomyocyte communication system and reducing the cardioprotective effect of IL-33 [87].

A good clinical practice is, however, a joint assessment of the changes of concentrations of several parameters. An assessment of the cardiovascular risk is possible with a higher sensitivity with simultaneous measurements of the concentrations of: sST2, hsTnI, hsCRP or GDF-15 [70]. The addition of serum galectin-3 concentration to that group is extremely useful in the case of disorders associated with myocardial dysfunction. It should be also mentioned that the biological variability of galectin-3 is low in comparison to other established and novel cardiovascular biomarkers (e.g., NT-proBNP, GDF-15, ST2) [19,88]. Apart from this, galectin-3 had 8.1% within-individual coefficient of variation in healthy controls and chronic HF patients [88]. Schindler et al. [89] presented that galectin-3 shown relatively low biological variability in healthy individuals and stable HF patients. Furthermore, among healthy subjects, galectin-3 had minimal biological variation in both the short- and long-term without sex differences.

Galectin-3 is a biomarker of ventricular remodelling and myocardial fibrosis [90]. It is also an important regulator of chronic and acute inflammatory condition and inflammation leading to fibrosis in various tissues [91]. The study by Tuegel et al. [92] demonstrates that simultaneously determined high concentrations of three biomarkers in serum: galectin-3, sST2 and GDF-15 in patients with chronic kidney disease are associated with a higher mortality of such patients. At the same time, it has been observed that only GDF-15 serum concentration is associated with development of heart failure. Serum galectin-3 concentration shows a low tissue specificity and, therefore, a multimarker strategy of cardiovascular risk assessment is needed [70]. Two studies involving patients with HF (CORONA and COACH trials) analysed the kinetics of changes in levels of serum galectin-3 after 0, 3 and 6 months [19,93]. The increase in serum galectin-3 concentration by ≥15% was found to indicate a 50% higher risk of mortality and hospitalization due to HF compared to patients with stable values of galectin-3 in the same time range, regardless of age, sex, diabetes, renal function, ejection fraction (LVEF) and NT-proBNP concentration. Moreover, the results of our study [81] demonstrated that patients with acute heart failure who died within 1 year of follow-up had significantly higher levels of serum galectin-3 at baseline compared to those who survived (55.6 ± 37.6 ng/mL vs. 15.0 ± 7.04 ng/mL; *p* = 0.005), which is in agreement with some previous reports. Our study was only a preliminary pilot research and the size of the study group is small.

In summary, it should be stressed that the proinflammatory effect of galectin-3 is not only limited to its participation in the pathogenesis of cardiovascular diseases. Recently, a significant importance of that marker has been demonstrated in the first place in SARS-CoV-2 infections. In patients with a severe COVID-19 course a systemic inflammatory condition develops, with intense cytokine storm, being the cause of respiratory failure and other multiple organ injuries [94,95]. It seems that serum galectin-3 concentration reflects the severity of COVID-19 course. In patients infected with SARS-CoV-2, in whom an unfavourable course of the disease forced administration of intensive therapy in view of respiratory failure, an almost three times higher serum galectin-3 concentrations were found, compared with patients not requiring treatment at intensive care units (ICUs) (23.46 (15.51–27.80) ng/mL vs. 8.93 (7.58–12.97) ng/mL, respectively) [63].

The study conducted by Kuśnierz-Cabala et al. [63] also called attention to the use of galectin-3 in the diagnosis of pneumonia, particularly in situations of severe COVID-19 course. In patients with pneumonia, an almost twice higher serum galectin-3 concentrations were found compared with those, in whom no pneumonia developed (13.30 (8.93–17.38) ng/mL vs. 8.55 (6.73–10.98) ng/mL, respectively). In that group of patients also higher concentrations were revealed of other proinflammatory markers: IL-6, pentraxin-3 (PTX-3), endothelial damage marker–soluble fms-like tyrosine kinase-1 (sFlt-1) and a number of tissue damage markers [63].

## 5. Therapeutic Potential of Galectin-3

A galectin-3-targeted therapy requires an understanding of the biology and pathologies associated with that molecule. Galectin-3 plays an important role in chronic inflammatory condition and is involved in the development of many diseases, including diseases of the heart [96,97,98], kidneys [99,100], viral infections [101], autoimmune diseases [102], neurodegenerative disorders [103,104,105] and many neoplastic diseases. In recent years a significant intensification has been observed of the studies on the role of galectin-3 as a central regulator of fibrosis and on therapeutic strategies aimed at inhibition of galectin-3 function in profibrotic diseases. The strategy of galectin-3 expression or function inhibition may be effective, not disturbing at the same time its normal main function.

Preclinical studies, in which a deletion of galectin-3-encoding gene was used, demonstrated the role of galectin-3 on the long list of cardiovascular diseases, including those focused on heart remodelling [106,107], hyperaldosteronism and arterial hypertension [108,109], acute myocardial infarction [110], myocardial ischemic/reperfusion injuries [111] and dilated cardiomyopathy [112]. That led to putting forward the hypothesis that galectins are potential targets for new anti-tumour and anti-inflammatory compounds. This hypothesis has been supported in several in vivo studies with the use of amino acid-derived lactulose-amine [113] and modified citrus pectin (MCP) [114]. The mentioned derivatives inhibit the expression of galectin-3. A development of small and large molecules blocking galectin-3 function may pave the way for a novel and exciting therapy with inhibitors, the action of which is, however, limited in healthy tissues.

### 5.1. Galectin-3 Inhibitors

#### 5.1.1. Monosaccharides

Pharmacological inhibition of galectin-3 with N-acetyllactosamine (N-Lac) prevented left ventricular dysfunction in heart-failure-susceptible REN2 rats [107] and also demonstrated a protective effect against hypertensive nephropathy in REN2 rats [115]. Low molecular mass saccharides, such as lactose or N-Lac, cannot be, however, used as “drugs” since they are rapidly absorbed and metabolised. Low molecular mass organic compounds, galactose derivatives, have also been synthesised and tested in respect of binding to the carbohydrate-binding domain of various galectins [116]. An inhibitor, 3,3′-ditriazolylthiodigalactoside exerted the strongest effect on galectin-3, with low values of the inhibition constant Kd = 29 nM [116].

#### 5.1.2. Galactomannans and Modified Citrus Pectins

Galactomannans (GMs) are plant-derived galectin-3 antagonists. GM-CT-01, known under proprietary name Davanat^®^ is a galactomannan with molecular mass 50 kDa and half-life from 12 h to 18 h [117,118]. Various types of modified citrus pectins with masses over 1000 kDa, e.g., GCS-100 [118] and PectaSol-C^®^ are already available on the market. The galectin-3-inhibiting effect of MCP was tested in various cell and animal models. The tests included: inhibition of haemaglutination by galectin-3, reduction of heart inflammatory condition, suppression of organ fibrosis and reduction of atherosclerosis in mice with apolipoprotein E deficiency [25,108,119]. The inhibiting effect of MCP on *LGALS3* gene expression was tested in cardiac fibroblasts in a rat experimental model of heart failure [120]. It was demonstrated [120] that MCP alleviated heart dysfunction, decreased the degree of myocardial damage and reduced collagen deposition. A reduction of *LGALS3*, TLR4, MyD88 gene expression and NF-κB-p65 factor inhibition were also described. A reduction was also observed of expression of IL-1β, IL-18, TNF-α– the proinflammatory cytokines involved in the pathogenesis of heart failure. The use of MCP as a galectin-3 antagonist exerted a favourable effect on myocardial dysfunction process through inhibition of inflammation and fibrosis. The use of galectin-3 as a potential therapeutic target in the treatment of heart fibrosis after infarction may bring many benefits [120]. It should also be said that in many in vivo studies no specificity of MCPs has been established, and it is possible that their inhibitory activity is caused by an effect also on other therapeutic targets.

#### 5.1.3. Thiodigalactosides

Recently, thiodigalactoside derivatives have been developed, with action targeted at new CRD sites, other than the canonical binding site. TD-139 is a thiodigalactoside analogue approved by the FDA for treatment of idiopathic pulmonary fibrosis, in the form of inhalation powder, and it has been speculated that it shows an effect antagonistic to galectin-3 through binding to B and E subsites [118,121]. TD-139 is a small molecule (C28H30F2N6O8S) of about 648 g/mol molecular mass and it can bind with a high affinity to both galectin-3 and galectin-1. Although the mechanism of action still remains unclear, it is supposed that the molecule allosterically modulates the CRD of galectin-3. Some research groups have also reported that thiodigalactosides can be preferentially adjusted to form more specific galectin-3 inhibitors [122].

#### 5.1.4. Heparin-Based Inhibitors

Heparin-based inhibitors are a relatively new and attractive group of galectin-3 inhibitors, which are sulphated or acetylated heparin derivatives. The results of in vitro studies have demonstrated that they are non-cytotoxic and selective for galectin-3 (i.e., they do not inhibit galectin-1, -4 and -8). The experimental in vivo studies with nude mice demonstrated that compounds induced by galectin-3 significantly inhibited the metastasizing of human melanoma and colonic cancer cells to the lungs. Moreover, the compounds showed no detectable anti-thrombotic activity and they seemed to be promising therapeutic agents [123]. They were, however, only tested in vivo models of metastases and, in the future, studies should be conducted in order to estimate their potential as anti-fibrotic agents.

#### 5.1.5. Neoglycoconjugates

Galectin-3 binds to branched saccharides with increased binding power, and large dendrimers connected with lactose as a functional group, provide an “excess of ligands” for galectin-3 binding. Michel et al. [124] studied the effect of various types of dendrimers, functionally bound to lactose, on tumour cell aggregation. They found that smaller dendrimers inhibited cell aggregation, possibly through competitive inhibition, while larger dendrimers containing several terminal lactose groups increased aggregation, providing thus many sites for galectin-3 binding [124]. Recently, yet other chemically modified glycoproteins (neoglycoproteins) have been developed, which show a potential to be used as new therapeutic molecules against fibrosis, through effective targeting at galectin-3. They serve not only as ligands with a high affinity, but can be also modulated in order to achieve selectivity for galectin-3, compared with other galectins. Their use in clinical context has not been assessed yet [125].

#### 5.1.6. Peptide Based Compounds

Amino-terminally truncated galectin-3 (Gal-3C) has been studied in the therapy of galectin-3-related tumours and seems to be a promising therapeutic target, showing a low toxicity profile [126,127]. There have been, however, no sufficient studies conducted, assessing its potential in the treatment of other galectin-3-associated disorders. Recently, Sun et al. [128] used the galectin-3-binding peptide i.e., G3-C12 for inhibition of intracellular galectin-3 in tumour cells. Since G3-C12 is highly selective for galectin-3 compared with other galectins, it acts as a selective galectin-3 target ligand. Then, when that peptide is coupled with the drug by means of a universal drug carrier, such as copolymer of N-(2-hydroxypropyl) methacrylamide (HPMA), the created G3-C12-HPMA-drug conjugate can easily penetrate the cells with galectin-3 over-expression [128]. The above-described concept of selective intracellular galectin-3 supplying can also find use in other scenarios of galectin-3-related diseases, such as organ fibrosis and HF.

Galectin-3 inhibitors are promising therapeutic agents, but still not much is known about their critical features, such as: in vivo power of action, absorption, metabolism, pharmacokinetics and toxicology. Although it has been demonstrated that they are active in some disease models, further studies are needed on the mechanism of their action, to establish whether they are active in a therapeutically acceptable model of supplying and dosing of the drugs for patients.

## 6. NcRNAs as the Modulators of *LGALS3* Gene Expression and a New Potential Therapeutic Target

The learning of the role of galectin-3 in the processes of myocardial fibrosis, inflammation and postinfarction dysfunction has also started studies, the aim of which was the search, at molecular level, for post-transcriptional mechanisms regulating *LGALS3* gene expression. The effect has been studied of short, non-coding RNAs, microRNAs (miRNAs), which participate in the regulation of many genes involved in such processes as: cell differentiation, division, proliferation, apoptosis and angiogenesis [129]. The mechanism of miRNAs action is based on the inhibition of protein translation process or direct degradation of mRNA of the target genes through binding of miRNAs to the complementary region of the target mRNA molecule [130]. Much data is available confirming the participation of miRNAs in the process of physiological regulation of the heart function and also in the progression of cardiovascular diseases [131,132,133,134].

The increased interest in circulating miRNAs as potential biomarkers of cardiovascular diseases has caused that successive studies started to appear, concerning the question whether miRNAs molecules can reflect the changes occurring at various stages of a pathological process. The correlation was studied between miRNAs specific to acute heart failure and the serum concentration of well-documented biochemical biomarkers including galectin-3 [135]. A negative correlation was demonstrated between miR-199a-3p expression and galectin-3 serum concentration in the 48th hour of hospitalisation in patients with impaired heart function and poor prognosis (r = −0.73; *p <* 0.001). The relationship observed between microRNA and established biomarkers, including galectin-3, can contribute to better elucidation of the role of unfavourable heart remodelling processes and fibrosis in the pathogenesis of acute heart failure. In study by Song et al. [136] *LGALS3* gene expression is increased in cardiac fibrosis process and over-expression of miR-199a expression has also been observed with hypertrophy heart failure. The biochemical and molecular biomarkers analysed can serve for predicting and better identification of patients with unfavourable prognosis in the case of impaired heart function [136,137].

The study by Zhang et al. [138] presented, in vivo and in vitro, using a murine experimental model, the role of miR-27b and explained some mechanisms underlying myocardial hypertrophy. In the case of miR-27b over-expression the heart function in the course of hypertrophy was restored, what suggested a protective role of miR-27b against the pathological process. The study demonstrated and experimentally confirmed that *LGALS3* was the target gene for miR-27b. Experimental *LGALS3* gene inactivation significantly suppressed the myocardial hypertrophy process. Both *LGALS3* and miR-27b have a potential as therapeutic and diagnostic targets in the treatment of cardiovascular diseases, including myocardial hypertrophy [138].

Increased *LGALS3* expression was also noted in the experimental murine model, in damaged cardiac tissue subjected to myocardial ischemic reperfusion (I/R) procedure [139]. On the other hand, an experimental silencing of *LGAL3* gene expression alleviated myocardial damage resulting from the I/R procedure. A bioinformatic prediction of the interactions between mRNA and miRNA demonstrated that the *LGALS3* was regulated by miR-204-5p. A reduced miR-204-5p expression level was observed in the cardiac tissue subjected to the I/R procedure. Moreover, a negative correlation was demonstrated between miR-204-5p expression and *LGALS* expression in the studied experimental system. Another important observation from the cited study [139] concerns the interaction between miR-204-5p and long non-coding RNA (lncRNA) *KCNQ1OT1*. A reduced *KCNQ1OT1* expression can provide protection against heart injury in the course of I/R after myocardial infarction, and *KCNQ1OT1* can modulate the expression of genes through a network of lncRNA/miRNA/mRNA interactions. *KCNQ1OT1*, binding to miR-204-5p, modulates the process of heart injury in the course of I/R through interaction with *LGALS3.* An increased *KCNQ1OT1* expression was noted in the heart tissue subjected to I/R, and a negative correlation was demonstrated between *KCNQ1OT1* expression and miR-204-5p expression in the studied experimental model. From the therapeutic point of view, the observation seems important, that experimental reduction of *KCNQ1OT1* expression and increase of miR-204-5p expression suppress cardiac injury in the course of I/R through a reduction of *LGALS3* expression. The presence of the network of *KCNQ1OT1*/miR-204-5p/*LGALS3* interactions may constitute a potential therapeutic target in the case of myocardial damage after I/R procedure [139].

*LGALS3* expression and influence of the lncRNA *SNHG20* and miR-335 regulatory factors were assessed in the process of myocardial fibrosis and hypertrophy induced by angiotensin II (Ang II) in a murine experimental model [140]. An increased *SNHG20* expression was noted, which was reflected in an increase of *LGALS3* expression, but a decreased miR-335 expression was observed in the cardiac tissue. An experimental loss of *SNHG20* function, which resulted in *SNHG20* expression reduction, caused a reduction of expression of the proteins involved in heart fibrosis and apoptosis processes and also increased the viability of the cells. An experimental over-expression or silencing of *SNHG20* through interaction with the miR-335/*LGALS3* system can modulate the cardiac fibrosis process induced by Ang II. *SNHG20* lncRNA can be another therapeutic target in the process of myocardial fibrosis and hypertrophy [140].

The diagnostic usefulness of the miR-1 and miR-21, known in the pathogenesis of heart failure, and of galectin-3 protein was analysed in a group of patients with acute heart failure and coexistent asymptomatic type 2 diabetes mellitus [141]. A negative correlation was found between miR-1 expression and galectin-3 serum concentration and a positive correlation was observed between miR-21 expression and serum galectin-3 concentration in the studied group of patients. An application of a panel of determinations including miRNAs and biochemical biomarkers can lengthen the odds on an early identification of patients with acute heart failure among patients with asymptomatic type 2 diabetes mellitus [141].

The participation of the mentioned miR-1, miR-21 and serum galectin-3 concentration was also studied in patients with symptomatic heart failure and left ventricular hypertrophy and history-confirmed arterial hypertension [142]. It was shown that in patients with heart failure a reduction of miR-1 and miR-21 expression occurred, and these results were significantly correlated with the concentration of serum galectin-3, the important factor playing the key role in the fibrotic process. The changes of miR-1 and miR-21 expression and serum galectin-3 concentration were analysed also in patients with systolic heart failure with various degrees of intensity of left ventricular dilatation [143]. A relationship was observed between reduced miR-1 expression with increased serum galectin-3 concentration and the progression of unfavourable heart remodelling, assessed as left ventricle dilatation. An increased miR-21 expression was also found in patients with decompensated systolic heart failure. In the study, it has been demonstrated that both up-regulation of miR-1 and miR-21 as well as galectin-3 lead to myocardial hypertrophy and unfavourable heart remodelling.

In the study by Han et al. [144], the clinical importance was assessed of miR-214 and serum galectin-3 concentration in whole blood of patients with chronic heart failure. A statistically significant increase was noted of both miR-214 expression and galectin-3 serum concentration in the group with chronic heart failure. A positive correlation was demonstrated between miR-214 expression and serum galectin-3 concentration, what suggested the participation of both factors in the development of chronic heart failure. Galectin-3 exerts an effect promoting the myocardial fibrosis process, while miR-214 can regulate fibroblast proliferation [144].

**Figure 3:**
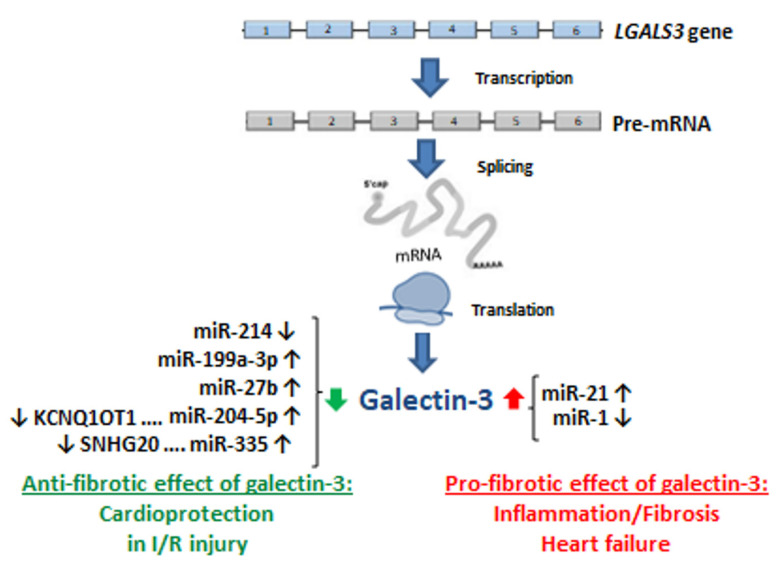
ncRNAs modulation of *LGALS3* gene expression in cardiovascular diseases. miR—microRNA; SNHG20—Small Nuclear RNA Host Gene 20; KCNQ1OT1—KCNQ1 Opposite Strand/Antisense Transcript 1. ↓—down-regulation; ↑—up-regulation; ….—interrelation between lncRNA and miRNA.

Galectin-3 can be not only a clinically important biomarker of fibrotic process in car-diovascular diseases, but also an interesting therapeutic target that can possibly decelerate the progression of heart fibrosis. lncRNAs and miRNAs are involved in the regulation of signalling pathways in cardiac fibroblasts. Expression silencing or over-expression of lncRNAs or miRNAs in vivo can prevent the fibrotic process and improve the diastolic heart function. The interrelations in the lncRNA/miRNA/Gal-3 axis can constitute a sus-ceptible target for therapeutic interventions, but further studies on this topic are required in order to increase the knowledge of their complex roles in the pathogenesis of cardiac fibrosis (Figure 3).

## 7. Conclusions and Perspectives

Based on the increasing literature data, galectin-3 emerges as a structurally unique and functionally extremely important galectin, expressed in various tissues and cell types and present not only inside but also outside cells, and also bound to cell membrane surfaces. The biological role of galectin-3 was initially ascribed to its carbohydrate-binding activity, but in the last decade a completely new spectrum of its functions was proved, not directly associated with the activity of that lectin. It has been found that galectin-3 participates in many pathological processes, in the first place in cardiovascular diseases but also in viral infections and many tumours.

Galectin-3 is an important factor in the pathophysiology of HF, mainly in view of its role in cardiac ventricular remodelling. Galectin-3 initially plays a protective role in the heart through its anti-apoptotic and anti-necrotic functions, while a prolonged expression of that protein leads to fibrosis and unfavourable remodelling of the damaged tissue. The sites of galectin-3 binding are mainly located in the extracellular matrix of the myocardium, fibroblasts and macrophages. Galectin-3 is released at the site of damage and activates the resting fibroblasts to become matrix-producing fibroblasts. The role of galectin-3 in fibroblast activation includes increased synthesis of cytoskeleton proteins, such as collagen type I, and inhibition of the activity of matrix metalloproteinases, what suggests that galectin-3 is involved in the initiation and development of the process of myocardial fibrosis. Usually, the expression of galectin-3 in a healthy heart is low, while its synthesis and release increase in fibrotic diseases, such as HF and AF. That creates wide possibilities of galectin-3 use in the diagnosis of cardiovascular diseases. Galectin-3 can provide additional information for the prognostication and stratification of HF risk. It seems, however, that the combination of biomarkers Galectin-3 and NT-proBNP or galectin-3 and cardiac troponins can provide a more precise clinical information than serum galectin-3 concentration alone. Apart from that use, galectin-3 seems to be a promising biomarker in cardiovascular diseases initiated and stimulated by inflammatory condition. It remains controversial whether this factor mediates unfavourable heart remodelling, or if it is merely a marker of heart failure. Some studies have provided evidence for a causal role of endogenous galectin-3 in the pathogenesis of myocardial fibrosis, hypertrophy and dysfunction in patients with heart failure. Based on these studies, it has been suggested that galectin-3 is not only a biomarker but also a mediator of the disease and it can be used as a therapeutic target [145].

Yu et al. [107] reported that the inhibition of galectin-3 was beneficial in mice that underwent transverse aortic construction (TAC) and in Ren2 hypertensive transgenic rats since it reduced fibrosis and improved function. Furthermore, in both murine and rat models of aldosterone-induced cardiac fibrosis, the loss of galectin-3 attenuated fibrotic changes and decreased cardiac dysfunction [108,109].

On the other hand, pharmacological and genetic inhibition of galectin-3 did not bring beneficial effects in a murine model of cardiomyopathy induced by transgenic activation of β_2_-adrenoceptors [42]. Both pharmacological and genetic inhibition occurred before the development of cardiomyopathy, and the obtained results suggest that the loss of galectin-3 cannot prevent, let alone, reverse the dysfunction in this experimental model. The sympathetic nervous system is activated in the course of cardiovascular diseases, which leads to enhanced and sustained stimulation of cardiac β-adrenoreceptors [146]. The activation of β-adrenoreceptors modulates the role of galectin-3 in heart disease, both as a biomarker and the mediator of the disease, as well as increases the level of circulating galectin-3 and directly regulates galectin-3 expression in the heart [147]. This could be the reason for obtaining different results depending on the experimental model and for attempts to undermine the role of galectin-3 as both an important mediator in the pathogenesis of CVD and a therapeutic target. In our opinion, galectin-3 plays a significant role in the pathogenesis of fibrotic heart disease as a factor stimulating the development of inflammation and worsening the prognosis. Thus, it seems that galectin-3 may be recognized not only as a biomarker but also as a promising therapeutic target. However, this thesis should be evaluated in further experimental and clinical research.

The role of galectin-3 in physiological and pathophysiological processes has inspired the development of its inhibitors as not only new therapeutic methods, but also as experimental tools for the basic sciences. These inhibitors may be useful in studying of the role of galectin-3 both in vitro (cell and tissue cultures) and in animal models, therefore they can contribute to the extension of the knowledge and better understanding of the intra- and extracellular functions of galectin-3.

The molecules of non-coding RNA (lncRNA and miRNA) play an important role in the regulation of *LGALS3* expression in various pathological conditions of the myocardium. These molecules can silence *LGALS3* expression at post-transcription level and thus can have an influence on the disease development. Moreover, an experimental over-expression of lncRNA and miRNA can prevent or alleviate the process of myocardial fibrosis. The learning of the lncRNA/miRNA/Gal-3 interrelations is very important, since they can constitute a target for therapeutic interventions. To achieve that goal an extension is needed of our knowledge of their complex role in the pathogenesis of heart diseases.

## Figures and Tables

**Table 1:** Various functions of galectin-3 in relation to cellular location.

Location	Function	References
Cytoplasm	Anti-apoptotic effect Proliferation	[6,7,11,12,13]
Nucleus	Gene transcription regulation pre-mRNA splicing promotion	[12,13]
Cell-surface	Diffusion and compartmentalization regulation Kinase and membrane receptors signalling	[14,15]
Extracellular environment	Cell adhesion Migration Growth regulation Pro-apoptotic effect	[16,17]

## Data Availability

Not applicable.
